# An ERP investigation of children and adolescents’ sensitivity to wins and losses during a peer observation manipulation

**DOI:** 10.1016/j.dcn.2021.100995

**Published:** 2021-07-29

**Authors:** Teena Willoughby, Taylor Heffer, Stefon van Noordt, James Desjardins, Sid Segalowitz, Louis Schmidt

**Affiliations:** a Brock University, Canada; b Montreal Neurological Institute, McGill University, Canada; c Montreal Neurological Institute, McGill University, Compute Ontario, Canada; d McMaster University, Canada

**Keywords:** Peer observation manipulation, Adolescence, Childhood, Pubertal status, ERP study, BART

## Abstract

The purpose of this ERP P3 study was to test a peer observation manipulation (being observed by a peer versus being alone) on neural markers of attention to reward (win-feedback) and punishment (loss-feedback) during the Balloon Analogue Risk Task. Participants (126 children, 53 % male, 8–10 years; 196 early adolescents, 50 % male, 11–13 years; and 121 mid-adolescents, 52 % male, 14–16 years) were assessed by age group and pubertal status. Individual differences in how participants felt about being observed by a peer, and self-report personality factors, also were examined. Findings indicated that early and mid-adolescents (and individuals in mid-puberty and late-puberty) were sensitive to peer observation as both groups showed larger neural responses to loss-feedback in the peer condition than in the alone condition. Conversely, children (and individuals in pre- and early-puberty) were unaffected by peer observation. In addition, there clearly were individual differences in how rewarding versus anxiety-provoking participants found the peer experience. Early adolescents and mid-adolescents (and individuals in mid- and late-puberty) who reported feeling more anxious about the peer observation elicited larger neural responses to loss-feedback, and individuals in mid- and late-puberty in particular reported higher worry and lower sensation-seeking scores than those who reported a positive experience.

## 1 Introduction

Adolescent risk taking is an important topic among both researchers and the general public (Dahl, 2004). Most risk taking occurs when adolescents are with their peers (Blakemore and Robbins, 2012; Steinberg, 2008). This finding, known as the peer effect, has been demonstrated in daily life (Doherty et al., 1998; Simons-Morton et al., 2011) and in lab experiments (e.g., Chein et al., 2011; Gardner and Steinberg, 2005). Even adolescents who are given only the *impression* that peers are observing them during a lab-based task have been found to engage in heightened risk taking relative to adolescents who complete the same task alone (Smith et al., 2014; Somerville et al., 2013, 2019; Weigard et al., 2014)

Recently, researchers have focused on how adolescent brain development might be implicated in the link between peers and risk taking (Smith et al., 2018; Steinberg, 2008; Telzer et al., 2013). For example, imbalance models suggest that there might be asynchrony in the maturation of neural circuitry within and between different brain systems, with circuitry within the subcortical limbic-striatal brain networks (associated with reward processing, including social rewards) maturing early in adolescence (likely due to puberty) but interconnections to the prefrontal executive networks (associated with self-control) maturing later in adolescence (Casey, 2015; Luna and Wright, 2016; Shulman et al., 2016; Steinberg, 2008, and Bjork et al., 2012; Crone and Dahl, 2012; Pfeifer and Allen, 2016, for criticisms of the model). This asynchrony in maturation is hypothesized to lead to heightened activation of the limbic-striatal networks during early to mid-adolescence, when neural connections to the prefrontal cortex networks that might dampen the activation (if appropriate) are not fully mature. Further, this asynchrony is thought to be most pronounced when adolescents experience high levels of emotional arousal, such as when adolescents are with their peers (Somerville et al., 2010).

The heightened activation of the limbic-striatal networks during puberty is thought to lead to increased reward seeking during adolescence in particular, including social rewards such as peer approval (Braams et al., 2015; Foulkes and Blakemore, 2016; Galván, 2010; Silva et al., 2017; Van Leijenhorst et al., 2010). Indeed, a growing literature supports a link between peer observation and heightened activation in limbic-striatal networks among adolescents but not adults (e.g., Chein et al., 2011; Smith et al., 2018), which suggests that adolescents find peers particularly rewarding (Smith et al., 2014, 2015; Weigard et al., 2014).

The limbic-striatal networks, however, are not exclusively sensitive to rewards. For example, activity in the ventral striatum and nucleus accumbens has been associated with both the gaining of social reward (e.g., peer approval) and the avoiding of social punishment (e.g., peer disapproval; Foulkes and Blakemore, 2016; Kohls et al., 2013; Levita et al., 2009). In fact, there likely are large individual differences in how adolescents are impacted by peer observation. While some adolescents may enjoy the presence of peers, others may worry about being negatively evaluated by their peers (Foulkes and Blakemore, 2018). In other words, some adolescents who are higher in sensation-seeking may find peers exciting while others who are more sensitive to threat and worry may find peers anxiety-provoking. Researchers examining the link between peer observation and adolescent risk taking, however, have not directly asked youth how they feel about the presence of peers in experimental sessions. We address this gap in the current peer observation study using event-related potentials (ERPs) to assess attention to reward and punishment while participants played the Balloon Analogue Risk Task (BART), either alone or when they thought they were being observed by a peer.

There has been some ERP research investigating general sensitivity to reward and punishment among children and adolescents using the BART. This work indicated that loss-feedback elicited larger neural responses than win-feedback (e.g., Euser et al., 2013; Yau et al., 2015). However, only one known peer observation study, involving 18 male adolescents, has assessed BART feedback processing using ERPs (Kessler et al., 2017). Consistent with findings by Euser et al. and Yau et al., Kessler and colleagues found greater attention to loss-feedback than win-feedback. Most importantly, compared to participants in the alone condition, participants in the peer condition showed larger neural responses only to loss-feedback. The authors speculated that one reason might be that losses are more salient than wins when being observed by a peer. We test this hypothesis in the current study by asking participants directly after playing the BART how they felt about having a peer watch them, and by focusing specifically on P3 activation, an ERP component that is typically larger when an individual is paying more attention to feedback (Huang et al., 2015; Luck, 2005). We chose to focus on the P3 as the findings related to the FRN have been mixed in the literature. In addition, several neuroimaging and EEG studies have shown that the FRN has been sourced to multiple brain regions, including dorsal anterior cingulate (Hauser et al., 2013), ventral prefrontal regions (Luu et al., 2007; Ruchsow et al., 2002; Segalowitz et al., 2010), and posterior cingulate and the basal ganglia (Walsh and Anderson, 2012). A comprehensive single subject analysis of frontal midline brain responses also revealed that the FRN sensitivity to feedback is only moderately robust across individuals (van Noordt et al., 2016). Thus, we felt that investigating the P3 as a marker associated with attention to feedback more closely aligned with our interest.

In addition, we include comparisons among children, early adolescents, and mid-adolescents to test the hypothesis that adolescence is a sensitive age period for social feedback processing. While experimental peer observation research generally compares adolescents to adults (Chein et al., 2011; Gardner and Steinberg, 2005; Segalowitz et al., 2012; Smith et al., 2014, 2015), studies comparing adolescents to children are lacking. Comparisons between early adolescents and mid-adolescents also are important given the potential role of puberty in the peer effect. Thus, we also compare how the results might differ depending on whether pubertal status or age is used to classify developmental differences. Importantly, neurodevelopmental imbalance models highlight that puberty might be a key reason for the brain changes that occur in adolescence (Casey, 2015; Somerville et al., 2010; see also Crone and Dahl, 2012). Furthermore, previous research has found that pubertal development may be a better marker than age (e.g., van den Bos et al., 2014).

### 1.1 The current study

The purpose of this ERP study is to test the peer effect (peer versus alone conditions) on neural markers of attention during reward (win) and punishment (loss) feedback processing during the BART. We address three main research questions: (1) Do participants in the peer condition exhibit larger P3 amplitudes to both win and loss feedback than participants in the alone condition? (2) Are there differences among age groups (children, early adolescents, and mid-adolescents) and among pubertal status groups (pre, early, mid and late puberty)? (3) Are there individual differences in how participants feel about being observed by a peer, and are they associated differentially with P3 amplitudes and personality self-report survey measures of sensation-seeking and worry?

Overall, we expect that participants in the peer condition will show larger neural responses than participants in the alone condition to loss-feedback but not win-feedback, given Kessler’s et al.’s (2017) findings. While the sensitive age period hypothesis for adolescents suggests that only early adolescents and mid-adolescents should exhibit the peer effect, it is not clear whether that hypothesis will be supported for children in the current study given the lack of research with that age group. We also expected that pubertal development might be a more sensitive measure than age. Finally, individual difference analyses in the peer condition are exploratory given the lack of research addressing this question.

## 2 Method

### 2.1 Participants

The current sample included 469 students (50.5 % female; age range: 8–15 years, grades 3–10) from several elementary and high schools in southern Ontario, Canada. Students were part of a larger longitudinal study examining the association between wellbeing and youth health-risk behaviors. Parent report indicated that 85.3 % of the children and adolescents were White, 2.3 % were Hispanic, 2.1 % were Black, 1.4 % were Asian, 0.9 % were Indigenous, and 7.4 % were Mixed Race (a further 0.7 % of parents indicated that they preferred not to answer the question). Mean levels of parental education fell between “completed an associate degree/diploma” and an “undergraduate degree”. Six participants were excluded from the study because they did not want to finish the BART task (one because of illness); 4 based on a diagnosis of autism, cerebral palsy, or epilepsy; 6 due to equipment issues; 1 due to removing many of the electrodes during the EEG task; and 9 participants because their data were not usable (e.g., contained a large number of muscle/movement artifacts). Thus, the final sample included 443 participants.

### 2.2 Procedure

Students were invited to participate in the study through visits to schools. Participants were tested at their school using a mobile lab trailer in the fall and spring and a quiet room in the school in the winter. Participants played the Balloon Analogue Risk Task (BART) while EEG was recorded. Participants were assigned randomly to complete the BART either alone or when they thought they were being observed by an anonymous peer (note that there was no peer watching). We explained to the participant that a student from another school was not able to do the BART task and wanted to see what it was like. We then pretended to phone that school (we called our lab manager instead) and confirm that the peer was able to see the participant’s computer. No students indicated that they did not believe the manipulation. After completing the BART task, participants in the peer condition were asked “How did you feel about playing for another student?” Given the size of the sample, EEG data collection occurred over two years. The survey measuring individual differences in sensation-seeking and worry was completed in students’ classroom during school hours (as part of the larger study), and all participants received gifts (e.g., backpacks) as compensation. The study received clearance from the University Research Ethics Board. Participants provided informed assent and their parents provided informed consent.

### 2.3 Missing data analysis

Behavioral data for the BART task was missing for eight participants (1.81 %) due to equipment problems. There also were missing data (1.39 %) for the survey questions (sensation-seeking and worry). Missing data for the survey (conducted at another time from the BART task) was primarily due to absenteeism from class on the day the survey was distributed. Missing data were imputed using the expectation-maximization algorithm (EM), using all study variables in the EM analysis (sex parental education, pubertal status, age). EM retains cases that are missing survey waves and thus avoids the biased parameter estimates that can occur with pairwise or listwise deletion (Schafer and Graham, 2002).

### 2.4 Measures

#### 2.4.1 Demographics

Sex and parental education (one item per parent, averaged together, using a scale of 1= did not finish high school to 6 = professional degree) were assessed.

#### 2.4.2 Age groups

Age of the participants was assessed. To distinguish between children and adolescents based on age group, 126 participants aged 8–10 years were considered children, 196 participants aged 11–13 years were considered early adolescents, and 121 participants aged 14–16 years were considered mid-adolescents.

#### 2.4.3 Pubertal status groups

Pubertal status was assessed using the Puberty Development Scale (PDS; Petersen et al., 1988). The PDS assesses body hair, facial hair, and voice development in boys, and body hair, menarche, and breast development in girls. All items were rated on a 4-point scale from 1 (*not yet started changing*) to 4 (*change seems complete*). For boys, their scores were summed such that a score of 3 was considered pre-puberty, a score of 4 or 5 (with no 3-point responses) was considered early puberty, a score of 6–8 (with no 4-point responses) was considered mid-puberty, and a score of 9 and over was considered late-puberty. For girls, a score of 2 and no menarche was considered pre-puberty, a score of three and no menarche was considered early-puberty, a score of 4 or more and no menarche was considered mid-puberty, and any score plus a yes to menarche was considered late-puberty (see Carskadon and Acebo, 1993 for scoring scheme). The PDS scale exhibits good reliability and validity (Carskadon and Acebo, 1993; Petersen et al., 1988). There were 66 pre-puberty, 76 early-puberty, 158 mid-puberty, and 143 late-puberty participants.

#### 2.4.4 Balloon analogue risk task

The Balloon Analogue Risk Task (BART) is a behavioral task that is used to measure risky decision-making (Lejuez et al., 2002). We used a modified version of the BART that is suitable for collecting ERPs (see Heffer and Willoughby, 2020). Specifically, participants were instructed to inflate a series of balloons in order to earn points. Participants indicated the number of pumps they wanted to inflate the balloon at the beginning of the trial (Euser et al., 2013; Pleskac et al., 2008; Yau et al., 2015). Participants then observed the balloon as it either safely reached the inflation number they picked (i.e., they won the points for that trial), or the balloon burst before reaching that point (i.e., they lost the points for that trial). Given that this task provides feedback associated with losing (i.e., when the balloon pops and points are lost) and winning (i.e., when the balloon does not pop and points are won), it facilitates the examination of sensitivity to rewards as well as sensitivity to punishment using ERPs (Chandrakumar et al., 2018; Fein and Chang, 2008; Gu et al., 2018; Takács et al., 2015).

The task consisted of 90 trials with a maximum breaking point of 20 pumps. The probability of the balloon popping increased as the number of pumps chosen increased (e.g., choosing to pump the balloon up to ‘15’ had a greater likelihood of it popping compared to pumping the balloon up to ‘5’). After feedback was presented, a new balloon appeared after 1000 ms. Participants earned one point for every pump of the balloon and points for all the “win” trials were summed to calculate their total points. No points were given for “loss” trials and points lost from each loss trial were not subtracted from the total number of points from win trials to calculate the final total. Participants were instructed that the goal of the task was to earn as many points as possible, as higher points would allow them to have better choices for the gift they received in compensation for completing the session. Participants also were told that the student observing them would receive the same gift.

#### 2.4.5 Sensation-Seeking

Sensation-seeking was assessed with four items (“I’ll try anything once”, I like doing things just for the thrill of it”, “When I go after something I use a “no fear” approach”, “I like trying new things”) on a scale ranging from 1 (Almost Never) to 4 (Almost Always). A principal components analysis indicated that these items formed one factor (factor loadings ranged from 0.73 to .79). Cronbach’s alpha was 0.75. As the EEG data collection occurred over two years (given the large sample size), participants’ sensation-seeking scores were taken from the survey of the corresponding year to their EEG session. Higher scores indicated higher levels of sensation-seeking.

#### 2.4.6 Worry

Worry was assessed with three items (“I know I should not worry about things but I just cannot help it”, “I worry about getting in trouble”, “I worry about making mistakes”) on a scale ranging from 1 (Almost Never) to 4 (Almost Always). A principal components analysis indicated that these items formed one factor (factor loadings ranged from 0.81 to .88). Cronbach’s alpha was 0.80. As the EEG data collection occurred over two years (given the large sample size), participants’ worry scores were taken from the survey of the corresponding year to their EEG session. Higher scores indicated higher levels of worry.

### 2.5 Electrophysiological recording and processing

Electroencephalography (EEG) was recorded continuously from a BioSemi ActiveTwo system using a 96-channel montage and 7 external sensors (zygomatic processes, outer canthi, inferior orbital bones, and one at the nasion). The data were digitized at a sampling rate of 512 Hz.

#### 2.5.1 Pre-processing

Pre-processing was performed using the EEG-IP Lossless pipeline (EEG-IP-L) to identify channels, independent components, and time course activity that reflected artifacts and relative non-stationarity (see Desjardins et al., 2021 for full details on this pipeline). During this procedure, the data were re-referenced and filtered with a 1 Hz (high pass) and 30 Hz (low pass) filter. Unreliable signals were identified using voltage variances across channels and time periods. The correlation between each channel and its three nearest neighbours was also used to assess unreliable signals.

Independent component analysis was then used to separate stable biological artifacts (e.g., heart rate components, eye blinks, EMG) from cortical source signals. Components were classified using the ICLabel plugin (Pion-Tonachini et al., 2019a, b). This process assesses each component against a crowd-sourced database to identify activation consistent with five different categories: eye blinks, neural, heart, lateral eye movements, muscle contamination, and mixed signal. After pre-processing, a manual quality control review was completed by a trained research assistant to validate pre-processing signal quality assessments based on component topographical projection, continuous activation, dipole fit and power spectrum profile. Full details of the pre-processing procedure used for this sample have been reported elsewhere: Heffer and Willoughby, 2020).

#### 2.5.2 EEG post-processing

**Fig. 1:**
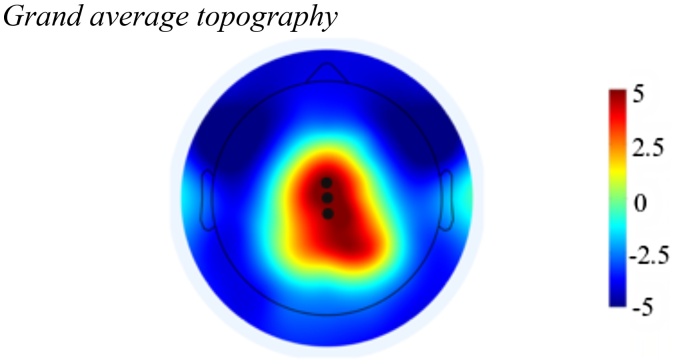
Grand average topography. Notes. Grand average topography at 350 ms (P3), collapsed across all participants, peer observation condition, and feedback type. Black dots indicate channel locations.

EEG data were then segmented into single trials and time-locked to the onset of the win/lose BART feedback stimuli. Epochs (−200 to 600 ms) were extracted to feedback onset and baseline corrected using the −200 to 0 ms pre-stimulus window. At this step, a final quality check was completed to identify and remove channels that had extreme voltage fluctuations (+/-50 μV). Channels that were removed during pre-processing were interpolated to reconstitute the full montage of 103 channels (96 scalp, 7 exogenous) using spherical spline. Similar to previous studies (Hassall et al., 2013; Kessler et al., 2017), the current study focused on central midline sites around Cz (electrodes A19, B19, and C7 in the Biosemi montage averaged together) to identify the P3 activation – see Fig. 1. The P3 was defined as the most positive peak within ∼300−400 ms post stimulus (e.g., Euser et al., 2011; 2013). Although some studies use a larger time window to capture the P3 (e.g., Fein and Chang, 2008; Gu et al., 2018; Kardos et al., 2016; Takács et al., 2015; Yau et al., 2015), based on visual inspection, our waveforms across each group had well-defined peaks that fell within the 300−400 ms time window.

### 2.6 Data analyses

Non-ERP data were statistically analyzed using IBM SPSS Statistics 25.0 software. ERP statistical analyses were carried out using STATSLAB, an open-source toolbox that implements robust statistics for analysis of EEG data (Campopiano et al., 2018). STATSLAB uses percentile bootstrap with trimmed means, techniques that are relatively insensitive to distribution characteristics such as skew, outliers, uneven tails, and various model assumption violations (see Wilcox, 2017). In STATSLAB, the three central midline sites were extracted as a region of interest and averaged together. For each participant, single trial data were re-sampled, with replacement, to generate a surrogate distribution. The 20 % trimmed mean across the surrogate trials was taken at each time point to generate an averaged robust ERP waveform (see Campopiano et al., 2018 for details). Significant differences were assessed against the 95 % confidence interval of the difference wave for a given categorical contrast. All ERP analyses were conducted first with condition (peer vs alone) as the between-subjects variable, type of feedback (win vs loss) as the within-subjects variable, and amplitude of the P3 as the dependent variable, across three age groups (children ages 8–10, early adolescents ages 11–13, and mid-adolescents ages 14–16). Analyses then were replicated again for pubertal status groups (pre-puberty, early-puberty, mid-puberty and late-puberty).

Behavioral data from the BART next were analyzed. We had five key variables of interest for the BART behavioral data: (1) total number of points earned, (2) total number of pumps, (3) reaction time after losses minus reaction time after wins (a positive reaction time suggests a longer reaction time to losses compared with wins, whereas a negative reaction time suggests a longer reaction time to wins compared with losses), (4) change in number of pumps (from the previous trial) after a loss, and (5) change in number of pumps (from the previous trial) after a win. ANOVAs were conducted with condition (peer vs alone) as the between-subjects independent variable and each behavioral data indicator as the dependent variable, first with age group as an additional between-subjects independent variable and then with pubertal status group. Sex and parental education were included in the analyses as covariates.

For participants in the peer condition, answers to the question “How did you feel about playing for another student?” were analyzed according to type of response (e.g., positive, anxious). Only response types that were provided from at least 10 % of the sample in each age group were included in further analyses. To investigate sensitivity to reward and punishment across the different response types, ERP analyses were conducted with response type as the between-subject independent variable, type of feedback (win vs loss) as the within-subject independent variable, and amplitude of the P3 ERP as the dependent variable, first including age group as an additional between-subjects independent variable and then with pubertal status group. Finally, we assessed the link between response type and participants’ scores on sensation-seeking and worry measures from the survey component of the study. MANOVAs were conducted with response type as the between-subject independent variable, and the sensation-seeking and worry survey scores as the dependent variables, first including age group as an additional between-subjects independent variable and then with pubertal status group. Sex and parental education were included in the analyses as covariates.

## 3 Results

A total of 223 participants were assigned randomly to the peer condition and 220 participants were assigned randomly to the alone condition. See Table 1 for the distribution of boys and girls across the age and pubertal status groups.

**Table 1:** Number of Boys and Girls in each Age and Puberty Group.

	Age Group			Pubertal Status			
	Children	Early Adol	Mid-Adol	Pre-Puberty	EarlyPuberty	Mid-Puberty	Late-Puberty
Boys	67	99	63	40	51	93	45
Girls	59	97	58	26	25	65	98

Note. Adol = Adolescents.

### 3.1 ERPs as a function of peer vs alone, wins vs losses, and age group vs pubertal status

#### 3.1.1 Age group analyses

**Fig. 2:**
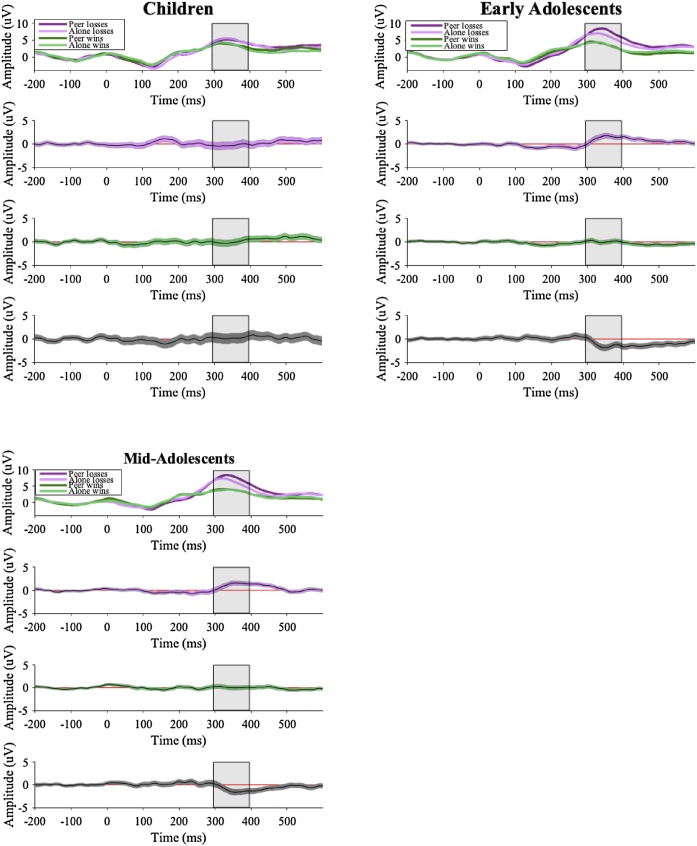
Waveforms for all age groups. *Notes*. Top panel shows waveforms for all age groups (the P3 is shown with the grey band). Second and third panels shows the 95 % bootstrapped confidence intervals for the differences between peer and alone conditions for losses (purple) and wins (green). Bottom panel shows the 95 % bootstrapped confidence intervals for the interaction between condition (peer vs alone) and type of feedback (losses vs wins). Time periods that show a red line depict a significant difference.

See Fig. 2 for results. Overall, participants had larger P3 amplitudes for losses than wins. For children, there was no difference in P3 amplitude between the peer and alone conditions for both loss-feedback and win-feedback. In contrast, for both early adolescents and mid-adolescents, there was a significant interaction between condition and type of feedback (wins vs losses). Consistent with our expectations, in both age groups there were larger P3 amplitudes for loss-feedback in the peer condition than in the alone condition. There was no significant difference, however, between the peer and alone conditions for win-feedback. In addition, P3 amplitudes for loss-feedback and win-feedback in the peer and alone conditions did not differ between early adolescent and mid-adolescent age groups, but both groups exhibited larger P3 amplitudes for loss-feedback than the children did.

#### 3.1.2 Pubertal status group analyses

**Fig. 3:**
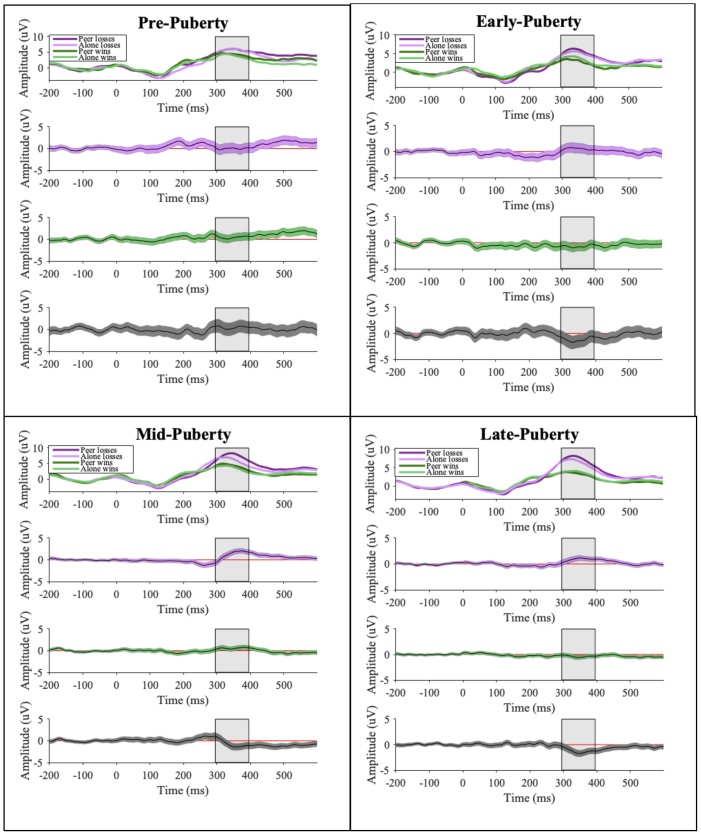
Waveforms for all puberty groups. *Notes*. Top panel shows waveforms for all puberty groups (the P3 is shown with the grey band). Second and third panels shows the 95 % bootstrapped confidence intervals for the differences between peer and alone conditions for losses (purple) and wins (green). Bottom panel shows the 95 % bootstrapped confidence intervals for the interaction between condition (peer vs alone) and type of feedback (losses vs wins). Time periods that show a red line depict a significant difference.

See Fig. 3 for results. Overall, participants had larger P3 amplitudes for loss-feedback than win-feedback. For pre-puberty, there was no difference in P3 amplitude between the peer and alone conditions for both loss-feedback and win-feedback. For early-puberty, mid-puberty and late-puberty, however, there was a significant interaction between condition and type of feedback. In the early-puberty group, there was a larger P3 amplitude for win-feedback in the alone condition than in the peer condition, while there was no significant difference between the peer and alone conditions for loss-feedback. In contrast, and consistent with our expectations, in both mid- and late puberty groups there were larger P3 amplitudes for loss-feedback in the peer condition than in the alone condition. There was no significant difference, however, between the peer and alone conditions for win-feedback. Furthermore, there were no differences in P3 amplitudes for losses in the peer and alone conditions between the pre- and early-puberty groups, while P3 amplitudes for win-feedback in the peer condition were larger in pre-puberty than early-puberty. P3 amplitudes for loss-feedback and win-feedback in the peer and alone conditions did not differ between mid- and late-puberty age groups (with the exception that P3 amplitudes for win-feedback in the peer condition were larger in mid-puberty than late-puberty), but both groups exhibited larger P3 amplitudes for loss-feedback than pre- and early-puberty groups.

### 3.2 BART behavioral results

On average, participants received win-feedback on 43.64 trials and loss-feedback on 46.36 trials. There was no difference between the peer vs alone conditions in the amount of win-feedback versus loss-feedback received, regardless of whether groups were selected using age group or pubertal status, *p*s > .05.

#### 3.2.1 Age group analyses

See Table 2 for means and standard deviations. For total number of points earned, there was a significant main effect for age group, *F*(2, 427) = 3.784, *p* = .024, η_p_^2^ = 0.017, with early adolescents having a higher number of points than children. There were no differences between the other age groups. For total number of pumps (a measure of risk taking), there was a significant main effect for age group, *F*(2, 427) = 3.290, *p* = .038, η_p_^2^ = 0.015, with mid-adolescents having a higher number of pumps than children. There were no differences between the other age groups. For reaction time, there was no significant difference. Regarding differences in change in number of pumps after loss-feedback or win-feedback, early adolescents and mid-adolescents were more likely than children to decrease the number of pumps following win-feedback, *F*(1, 427) = 3.166, *p =* .043, η_p_^2^ = .015, the peer group was more likely than the alone group to decrease the number of pumps following win-feedback, *F*(1, 427) = 7.983, *p =* .005, η_p_^2^ = .018, decrease the number of pumps following loss-feedback, *F*(1, 427) = 5.127, *p* = .024, η_p_^2^ = .012, and increase the number of pumps following loss-feedback, *F*(1, 427) = 5.654, *p* = .018, η_p_^2^ = .013 - in other words, the latter two results indicate that the peer group was more likely than the alone group to change the number of pumps, either way, following loss-feedback. There were no other significant differences.

**Table 2:** Means and standard deviations for behavioral results by age group.

	Peer			Alone		
	Children	Early Adolescents	Mid-adolescents	Children	Early Adolescents	Mid-adolescents
Total Points	325.46 (62.99)	353.36 (59.83)	346.12 (55.89)	328.68 (61.53)	337.06 (54.74)	326.69 (46.66)
Total Pumps	867.46 (217.33)	926.08 (166.61)	920.26 (188.70)	883.13 (193.37)	882.09 (168.81)	951.09 (167.23)
Reaction Time	−19.31 (415.46)	−23.94 (338.77)	−6.62 (267.45)	−113.80 (517.42)	−48.87 (312.43)	−37.59 (478.21)
Loss Decrease	32.76 (7.01)	33.30 (8.31)	33.93 (8.40)	31.90 (9.84)	30.96 (7.50)	31.54 (8.77)
Loss Increase	50.58 (9.60)	51.34 (11.23)	52.81 (11.55)	46.77 (10.51)	50.53 (10.66)	49.90 (12.53)
Loss No Change	16.66 (12.76)	15.36 (15.10)	13.26 (13.96)	21.33 (16.22)	18.51 (13.92)	18.56 (16.56)
Win Decrease	47.96 (9.53)	50.10 (11.38)	51.19 (10.15)	44.72 (11.28)	47.77 (10.66)	47.91 (11.68)
Win Increase	32.42 (9.47)	32.92 (8.77)	33.36 (9.75)	30.37 (9.02)	32.93 (9.16)	34.25 (10.07)
Win No Change	19.62 (13.29)	16.98 (15.52)	15.45 (13.90)	24.91 (15.10)	19.30 (13.78)	17.84 (16.87)

Notes. The mean percentage of decreases and increases after a loss or win are shown (percentages are out of the total number of losses or wins). Note that the ‘no change’ category for losses and wins are shown but not analyzed given their dependency (and thus redundancy) with the number of decreases and increases. A negative reaction time indicates a longer reaction time to wins compared with losses.

#### 3.2.2 Pubertal status analyses

See Table 3 for means and standard deviations. There was a significant interaction between condition (peer vs alone) and pubertal status in the number of total points gained during the BART task, *F*(3, 425) = 4.030, *p* = .008, η_p_^2^ = 0.028.

**Table 3:** Means and standard deviations for behavioral results by pubertal status.

	Peer				Alone			
	Pre	Early	Mid	Late	Pre	Early	Mid	Late
Total Points	314.15 (65.49)	360.10 (60.51)	349.69 (59.94)	340.74 (54.31)	341.99 (56.59)	323.00 (56.09)	330.90 (54.76)	333.28 (53.93)
Total Pumps	819.44 (202.96)	925.90 (182.82)	924.92 (168.05)	921.07 (201.21)	879.66 (166.95)	865.19 (177.62)	918.32 (198.44)	906.27 (157.74)
Reaction Time	4.84 (512.12)	−13.94 (337.95)	−19.18 (325.04)	−28.90 (260.01)	−179.48 (624.99)	−68.32 (419.56)	−37.01 (430.82)	−42.17 (288.47)
Loss Decrease	33.41 (7.35)	32.21 (8.33)	34.36 (7.58)	32.76 (8.48)	29.28 (9.78)	31.13 (8.61)	31.39 (7.53)	32.40 (8.89)
Loss Increase	50.97 (9.87)	48.68 (10.57)	51.33 (10.56)	53.78 (11.63)	45.83 (13.65)	49.90 (9.78)	50.35 (10.24)	49.34 (11.57)
Loss No Change	15.62 (12.50)	19.11 (15.78)	14.31 (13.25)	13.46 (14.69)	24.89 (19.45)	18.97 (14.39)	18.26 (12.24)	18.26 (16.35)
Win Decrease	48.74 (9.69)	47.10 (11.98)	49.47 (10.54)	52.33 (9.74)	44.96 (12.37)	46.25 (11.37)	48.49 (10.57)	46.46 (11.10)
Win Increase	32.78 (8.91)	31.10 (11.17)	33.79 (8.821)	33.00 (8.64)	29.28 (11.49)	30.33 (7.41)	32.97 (8.18)	34.49 (10.13)
Win No Change	18.48 (13.59)	21.80 (17.23)	16.74 (13.46)	14.67 (13.86)	25.76 (17.03)	23.42 (15.74)	18.54 (12.78)	19.05 (16.01)

Notes. The mean percentage of decreases and increases after a loss or win are shown (percentages are out of the total number of losses or wins). Note that the ‘no change’ category for losses and wins are shown but not analyzed given their dependency (and thus redundancy) with the percentage of decreases and increases. A negative reaction time indicates a longer reaction time to wins compared with losses.

Specifically, early puberty and mid-puberty groups in the peer condition gained higher total points than the early puberty and mid-puberty groups in the alone condition, *F*(1, 71) = 5.679, *p* = .020, η_p_^2^ = .074 for early puberty, and *F*(1, 150) = 4.145, *p* = .044, η_p_^2^ = .027 for mid-puberty. For total number of pumps (a measure of risk taking), there was a significant main effect for pubertal status, F(3, 425) = 2.838, p = .038, η_p_2 = 0.020, with both mid- and late-puberty groups having a higher number of pumps than the pre-puberty group. There was no significant difference for reaction time. Identical to the analyses using age group as a between-subjects factor, the peer group was more likely than the alone group to decrease the number of pumps following win-feedback, *F*(1, 425) = 6.946, *p =* .009, η_p_^2^ = .016, decrease the number of pumps following loss-feedback, *F*(1, 425) = 6.056, *p* = .014, η_p_^2^ = .014, and increase the number of pumps following loss-feedback, *F*(1, 425) = 4.394, *p* = .037, η_p_^2^ = .010 - in other words, the latter two results indicate that the peer group was more likely than the alone group to change the number of pumps, either way, following loss-feedback.

### 3.3 Qualitative data analyses in peer condition

For participants in the peer condition, answers to the question “How did you feel about playing for another student?” were analyzed according to type of response. Only two types included responses from at least 10 % of the sample in each age group.

One type was positive statements; for example:

• “Good, especially when I got all those 10s”
• “They must be really happy with me. I hope they are happy with how I’m doing”
• “Fine, it wasn’t a big deal”
• “It was fun”
• “Good”

The other type was statements indicating anxiety about the peer observation, for example:

• “I felt stressed out. I didn’t want to let them down”
• “Nerve-wracking, because I felt like if I failed, they were going to think I failed them too”
• “I felt a lot of pressure. Like, around the end there, I just kept pressing and they kept exploding and I was thinking "oh no!"
• “It was kind of a lot of pressure”
• "Interesting…(hesitating) But I felt kind of embarrassed when I was losing."
• “I felt kind of nervous”.

Given the low n for anxious responses with the younger groups, we collapsed groups that showed similar patterns of P3 amplitude (i.e., pre- and early-puberty groups, mid- and late-puberty groups, and early adolescent and mid-adolescent groups). See Table 4 for details on the type of responses and percentages by age group and by pubertal status. Descriptively, positive statements were more prevalent with younger groups than older and more advanced pubertal status groups, while the opposite was true for anxious statements.

**Table 4:** Percentages of qualitative responses in the peer condition.

Responses by Age Group	Children Percentage (n)	Early Adolescents/Mid-Adolescents Percentage (n)
Positive	71.0 (44)	49.1 (79)
Anxious	19.4 (12)	26.1 (42)
“It was weird”	3.2 (2)	6.8 (11)
“I don’t know”	0.0 (0)	6.8 (11)
“I forgot that someone was watching”	1.6 (1)	6.2 (10)
Other – was not relevant to question	0.0 (0)	3.1 (5)
No response	3.2 (2)	1.2 (2)
RA forgot to ask the question	3.2 (2)	0.6 (1)

**Responses by Pubertal Status**	**Pre/Early Puberty Percentage (n)**	**Mid/Late Puberty Percentage (n)**

Positive	66.7 (46)	50.0 (77)
Anxious	18.8 (13)	26.6 (41)
“It was weird”	2.9 (2)	6.5 (10)
“I don’t know”	1.4 (1)	6.5 (10)
“I forgot that someone was watching”	2.9 (2)	5.8 (9)
Other – was not relevant to question	1.4 (1)	2.6 (4)
No response	2.9 (2)	1.3 (2)
RA forgot to ask the question	2.9 (2)	0.6 (1)

Note. Response percentages to the question “How did you feel about playing for another student”?

#### 3.3.1 ERP results for qualitative responses in peer condition

**Fig. 4:**
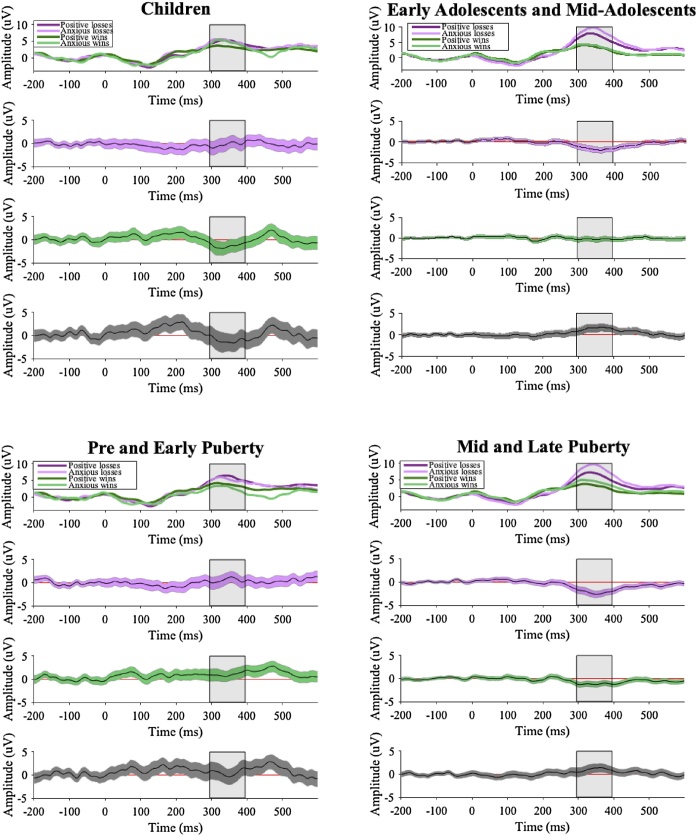
Waveforms for all qualitative groups. *Notes*. Top panel shows waveforms for all qualitative groups (the P3 is shown with the grey band). Second and third panels shows the 95 % bootstrapped confidence intervals for the differences between peer and alone conditions for losses (purple) and wins (green). Bottom panel shows the 95 % bootstrapped confidence intervals for the interaction between condition (peer vs alone) and type of feedback (losses vs wins). Time periods that show a red line depict a significant difference.

See Fig. 4 for results investigating age group and pubertal status differences in neural sensitivity to loss-feedback and win-feedback across the positive and anxious response types in the peer condition. For children, there was no significant difference between the positive and anxious groups in P3 amplitude for loss-feedback, but the anxious group had larger P3 amplitudes for win-feedback than the positive group. In contrast, for participants in the combined early adolescent and mid-adolescent group, there was a significant interaction whereby there were larger P3 amplitudes for the anxious group than the positive group for loss-feedback, but not for win-feedback. For the combined pre- and early-puberty group, there was no significant difference between the positive and anxious groups in P3 amplitude for loss-feedback or win-feedback. In contrast, for participants in the combined mid- and late puberty group, there was a significant interaction whereby there were larger P3 amplitudes for the anxious group than the positive group for both loss-feedback and win-feedback, with the difference larger for loss-feedback than win-feedback.

#### 3.3.2 Link between positive and anxious responses and survey measures of sensation-seeking and worry

Results of a MANOVA with positive vs anxious response type and age group as the independent variables, and sensation-seeking and worry as the dependent variables, indicated no significant effects, Wilks λ(2, 150) = 1.946, *p* = .146, η_p_^2^ = .025 for the interaction between response type and age group. In contrast, for the pubertal status analysis, there was a significant main effect for pubertal status, Wilks λ(2, 150) = 5.862, *p* = .004, η_p_2 =.072, specifically for worry. The pre-early puberty group reported lower worry scores on the survey (*M* = 2.66, *SD* = 0.73) than the mid-late puberty group (*M* = 2.32, *SD* = 0.76), *F*(1, 156) = 1.980, *p* = .001, η_p_^2^ =.071. There also was a significant interaction between positive vs anxious response type and pubertal status, Wilks λ(2, 150) = 3.955, *p* = .021, η_p_^2^ =.050. For the combined mid-late puberty group only, the positive group reported higher scores on the sensation-seeking survey measure (*M* = 2.92, *SD* = 0.61) than the anxious group (*M* = 2.50, *SD* = 0.62), *F*(1, 103) = 11.647, *p* = .001, η_p_^2^ =.104, and the anxious group reported higher scores on the worry survey measure (*M* = 2.833, *SD* = 0.683) than the positive group (*M* = 2.45, *SD* = 0.61), *F*(1, 103) = 4.398, *p* = .038, η_p_^2^ =.042.

## 4 Discussion

Much of the past literature on the peer effect has been based on adolescent samples or on comparisons between adolescents and adults (e.g., Chein et al., 2011; Segalowitz et al., 2012; Smith et al., 2018). The goal of the current study was to investigate the peer effect among children, early adolescents, and mid-adolescents (and different pubertal statuses). Critically, there was a significant peer effect starting already in early adolescence (and mid-puberty), with participants in the peer condition showing larger neural responses to loss-feedback than participants in the alone condition, regardless of whether adolescence was characterized by age or pubertal status. In contrast, feedback processing in children (and pre- and early puberty) was not affected by peer observation. These findings support previous research suggesting that adolescence represents a sensitive period for social processing (Blakemore and Mills, 2014).

Of interest, our study found that the peer effect was specific to loss-feedback, not win-feedback (see also Kessler et al., 2017). Researchers interested in the imbalance model often highlight adolescence as an age period for *reward* sensitivity (e.g., Chein et al., 2011; Shulman et al., 2016; Smith et al., 2018). Yet, subcortical regions implicated in the imbalance model (e.g., the ventral striatum, nucleus accumbens) are activated not only to social reward (e.g., peer approval) but also to social punishment (e.g., peer disapproval; Foulkes and Blakemore, 2016; Kohls et al., 2013; Levita et al., 2009). In fact, Foulkes and Blakemore (2016) argue that adolescents show hypersensitivity to all social stimuli, not just positive or rewarding stimuli. Our results support that suggestion in that adolescents in the current study focused more on punishment (i.e., loss-feedback) than reward (i.e., win-feedback).

The finding that youth are more sensitive to loss-feedback compared to win-feedback also is in line with previous ERP studies using the BART (e.g., Euser et al., 2013; Yau et al., 2015); however, it is not entirely consistent with the Imbalance Model, which would suggest that adolescents should also be sensitive to rewards, especially in the context of peers. It is not clear *why* youth are sensitive to loss-feedback but not win-feedback. Kessler et al. (2017) speculate that losing points because you played too risky may be more disappointing than the thrill of winning points when you played it safe. Losing points on a trial provides clear feedback that you might have made a poor decision (i.e., they were too risky). Winning points on a trial, however, may suggest that you made the right choice *or* it could mean that you missed an opportunity to earn even more points if you had been riskier; thus, win feedback may not be as rewarding in this situation.

There also may be an important developmental shift in attention to loss feedback, particularly in the context of peers. Indeed, adolescence is a time in which peers, rather than parents, have more influence on social behavior (Steinberg and Silverberg, 1986). For example, adolescents are more likely to be influenced by the opinions of other teenagers, while children and adults are more likely to be influenced by the opinions of adults (Blakemore, 2018). Adolescents, compared to children and adults, also report more embarrassment and have higher skin conductance during peer observation (Somerville et al., 2013). Further, adolescents tend to report feeling worse about peer rejection than other age groups (e.g., O’Brien and Bierman, 1988; Sebastian et al., 2010). Taken together, these findings suggest that (1) adolescents have heightened sensitivity to peers and (2) contexts that have the potential to result in peer rejection or disapproval are particularly concerning for adolescents (Blakemore and Mills, 2014). Thus, receiving negative feedback in front of a peer would be a more salient and concerning event for adolescents than children.

To further investigate this peer effect, we asked participants how they felt about playing the BART while a peer observed. Although many participants had a positive experience of playing for a peer (e.g., “Good, especially when I got all those 10s”), others found that playing for a peer was anxiety provoking (e.g., “Nerve-wracking, because I felt like if I failed, they were going to think I failed them too”). Positive statements were more common among children (and individuals in pre-early puberty) than early and mid-adolescents (and individuals in mid-late puberty), while for anxious statements the reverse was true (i.e., more common among early and mid-adolescents and individuals in mid-late puberty). Among the early and mid-adolescents (and individuals in mid-late puberty), participants who were anxious about the peer observation during the BART had greater sensitivity to loss-feedback than participants who reported a positive experience. Thus, there are important individual differences among adolescents — not all adolescents find peer observation rewarding. This finding confirms that attention to social context is critical when studying adolescents (Crone and Dahl, 2012; Defoe et al., 2015).

Interestingly, the positive groups also paid more attention to losses than wins (as demonstrated with the P3 results), although not to the same extent as the anxious groups. It is not entirely clear why individuals who reported that they enjoyed playing the BART in front of a peer would be sensitive more to loss-feedback than win-feedback. It could be that loss-feedback in general is more salient than win-feedback — in line with Kahneman and Tversky (1979) who suggested that “losses loom larger than gains.” It also could be that this group had a fun but competitive experience (e.g., “Good, especially when I got all those 10s”) and so losing points on trials would also bother them.

An important strength of this study was that we used both pubertal status and age as indicators of development. Pubertal development and age are highly correlated in this study (*r* = .74) and we found many consistencies between these measures in our findings. Regardless of whether it was classified by age or pubertal status, adolescents had a higher number of pumps (an indicator of risk taking) than children. At the same time, age and pubertal status are not completely synonymous indicators of development. In fact, there is considerable variability in the age at which individuals reach puberty (e.g., Berenbaum et al., 2015). We found some differences depending on whether age or pubertal status was used. Most notably, we found that only for pubertal status analyses, early- and mid-puberty groups in the peer condition had a higher number of points in the BART than early- and mid-puberty groups in the alone condition. Further, for the mid-late puberty group (not the early and mid-adolescent group), we found that the positive group reported higher scores on the sensation-seeking survey measure than the anxious group, while the anxious group reported higher scores on the worry survey measure than the positive group. These findings were not significant when we used age to classify the groups. It could be that pubertal status is a more sensitive measure to capture individual differences in peer sensitivity. Indeed, the imbalance model highlights the role of puberty in particular as a key measure associated with adolescent brain development (Casey, 2015; Somerville et al., 2010). In line with this idea, pubertal development in particular has been found to be associated with both sensation seeking/reward sensitivity (e.g., Steinberg et al., 2008; Urošević et al., 2014) and anxiety/threat sensitivity (e.g., Nelson et al., 2005; Stenson et al., 2020), consistent with the findings related to sensation-seeking and worry in the current study. At the same time, further research is needed to help clarify the unique role of puberty in comparison to age (especially in light of their high correlation). For example, while age changes linearly across adolescence and the change is consistent for all youth, there are important individual differences in the rate at which adolescents go through pubertal development, which could be important to measure and take into account in analyses.

Overall, this study has a number of important strengths that have yet to be highlighted. First, we used a large ERP sample of children, early adolescents, and mid-adolescents. A large sample was critical in order to investigate participants’ qualitative responses in the peer condition. Indeed, asking participants in the peer condition about how they felt playing for a peer revealed important individual differences. Second, although the peer effect has become a topic of great interest among developmental researchers, this effect often is identified only by studying adolescents or by comparing adolescents to adults. Our study adds an important contribution by identifying this adolescent-specific peer effect when comparing children and adolescents. Finally, our robust tests for ERP differences illustrate significant differences using confidence intervals around the time course of effects and is more informative (e.g., showing variability) than solely relying on p-values.

Despite these strengths, our study is not without limitations. First, it would have been beneficial to include an adult sample in order to capture developmental differences in the peer effect across the lifespan. It would be interesting to see how adults qualitatively respond to playing in front of a peer. Second, access to longitudinal data would be preferable in order to investigate how the peer effect may change within a person as they transition from childhood to adolescence. Nonetheless, our study has important implications pertaining to developmental differences in the peer effect among children and adolescents.

### 4.1 Conclusions

Our findings suggest that sensitivity to the impact of peer observation during a risk-taking task begins to emerge in early adolescence (and mid-puberty). Both early adolescent and mid-adolescent groups (as well as mid- and late-puberty groups) had larger attention-related ERPs to loss-feedback in the peer condition than in the alone condition. Children (and pre- and early-puberty groups), on the other hand, were unaffected by peers observing them. At the same time, there clearly were individual differences in how rewarding versus anxiety-provoking adolescents found the peer experience. Early adolescents and mid-adolescents (as well as mid- and late-puberty groups) who reported feeling more anxious about the peer observation paid greater attention to loss-feedback than those who were more positive about the peer observation. Interestingly, the mid- and late-puberty anxious group (but not the early- and mid-adolescent anxious group) reported higher worry but lower sensation-seeking scores than those who reported a positive experience. Although results were most often consistent regardless of whether groups were analysed by age or pubertal status, differences in the results suggest that pubertal status might be a more sensitive measure than age in capturing individual differences in sensitivity to peer observation.

## Data statement

As the data for this study are part of an ongoing longitudinal study, data currently are not available to access.

## Declaration of Competing Interest

The authors declare that they have no known competing financial interests or personal relationships that could have appeared to influence the work reported in this paper.
